# Digital and Hybrid Pediatric and Youth Mental Health Program Implementation Challenges During the Pandemic: Literature Review With a Knowledge Translation and Theoretical Lens Analysis

**DOI:** 10.2196/55100

**Published:** 2024-06-25

**Authors:** Lynnette Lyzwinski, Sheila Mcdonald, Jennifer Zwicker, Suzanne Tough

**Affiliations:** 1 Department of Paediatrics Cumming School of Medicine University of Calgary Calgary, AB Canada; 2 The School of Public Policy University of Calgary Calgary, AB Canada; 3 Department of Community Health Sciences University of Calgary Calgary, AB Canada

**Keywords:** mental health, knowledge translation, KT, flourishing, youth, teenagers, mindfulness, positive psychology, telehealth, implementation, knowledge dissemination, pandemic, COVID-19, service delivery

## Abstract

**Background:**

The pandemic brought unprecedented challenges for child and youth mental health. There was a rise in depression, anxiety, and symptoms of suicidal ideation.

**Objective:**

The aims of this knowledge synthesis were to gain a deeper understanding of what types of mental health knowledge translation (KT) programs, mental health first aid training, and positive psychology interventions were developed and evaluated for youth mental health.

**Methods:**

We undertook a literature review of PubMed and MEDLINE for relevant studies on youth mental health including digital and hybrid programs undertaken during the pandemic (2020-2022).

**Results:**

A total of 60 studies were included in this review. A few KT programs were identified that engaged with a wide range of stakeholders during the pandemic, and a few were informed by KT theories. Key challenges during the implementation of mental health programs for youth included lack of access to technology and privacy concerns. Hybrid web-based and face-to-face KT and mental health care were recommended. Providers required adequate training in using telehealth and space.

**Conclusions:**

There is an opportunity to reduce the barriers to implementing tele–mental health in youth by providing adequate technological access, Wi-Fi and stationary internet connectivity, and privacy protection. Staff gained new knowledge and training from the pandemic experience of using telehealth, which will serve as a useful foundation for the future. Future research should aim to maximize the benefits of hybrid models of tele–mental health and face-to-face sessions while working on minimizing the potential barriers that were identified. In addition, future programs could consider combining mental health first aid training with hybrid digital and face-to-face mental health program delivery along with mindfulness and resilience building in a unified model of care, knowledge dissemination, and implementation.

## Introduction

### Background

The COVID-19 pandemic and resultant closures of schools during lockdowns worldwide brought on major challenges for child and youth mental health [1-3]. A systematic review found that, as a result of school closures, there was a rise in depression, anxiety, emotional and behavioral problems, stress, and suicidal attempts in children and teenagers during the pandemic period [4]. Many teenagers reported challenges with coping with their mental health due to isolation from staying at home and a lack of social contact with peers [2]. In particular, there were unique challenges for children and teenagers with existing mental health problems when it came to accessing timely basic mental health care services during the pandemic. This was due to pandemic-related closures of medical clinics [5] and school-based health services [2], limited capacity of medical doctors, and prioritization of patients with COVID-19, which left many patients with chronic health problems with lower levels of accessibility to care [6]. A study in the United Kingdom found that 26% of teenagers felt that they had reduced access to mental health care [1]. Furthermore, a study in Italy found reduced emergency department admissions for mental health problems as patients remained at home and socially isolated distanced themselves to reduce their risk of infection [7]. However, it should be noted that not all studies found that everyone had been equally impacted by COVID-19 and had experienced mental health challenges, with some studies finding improvements in some individuals, particularly those without a preexisting mental health condition [8]. Nevertheless, child and youth mental health was a critical public health challenge during the pandemic for many.

As a result of the mental health crisis in youth during the pandemic, medical doctors had to quickly transition to digital tele–mental health care services to meet the pressing mental health needs of children and teenagers and increase their accessibility to care [9]. Many service providers had to implement digital mental health care for the first time, which came with its unique challenges [9]. Before the COVID-19 pandemic, tele–mental health was less prevalent and often used in rural and remote patient settings [10]. However, as medical providers adjusted to the “new normal,” tele–mental health and other digital mental health approaches became more mainstream [11]. Nevertheless, it is also important to examine what other mental health programs, including face-to-face or combined digital programs, were implemented during the pandemic to better understand differences in program preferences and experiences.

Understanding the key challenges and experiences with these different approaches and models of psychiatric mental health care is essential to make recommendations for future best practice guidelines and pandemic preparedness. Understanding the facilitators of effective implementation of youth mental health services in clinical medical settings and how to overcome barriers is necessary for making recommendations for effective tele–mental health and in-clinic program implementation. Moreover, it is important to understand implementation challenges, including barriers to implementation, considering determinant frameworks that assess barriers to implementation rather than solely examining structural process models of implementation that describe how the program was implemented and the specific procedural steps [12]. Analyzing studies that used both structural and process frameworks and the models used when implementing studies during the pandemic will provide deeper insights into what was specifically developed, the processes that were undertaken, and the challenges that were experienced.

In addition to implementing psychiatric programs in clinical settings that are administered through clinical mental health professionals, there is also a need to better understand what mental health programs were implemented in the community setting, such as in schools through school-based officials and in charities, and understand and what efforts by key stakeholders to promote youth mental health and build resilience in children and youth (preteenagers, teenagers, and young adults). It is of particular interest to investigate whether stakeholders working to support children (eg, community-based organizations such as the YMCA) and mental health–specific organizations implemented youth mental health programs during the pandemic in schools and the general community setting. It is also of interest to understand any key program implementation challenges to make recommendations for implementation and policy research.

Furthermore, there is a need to evaluate the barriers to and facilitators of psychological programs, including self-guided ones in the home setting that taught youth how to manage their emotions during isolation and times of crisis and uncertainty. In particular, positive psychology and especially mindfulness-based approaches have been found to foster greater gratitude and well-being in children and youth [13-15], and it is of interest to evaluate what approaches were used to assist youth during pandemic times of crisis from the perspective of adoption and uptake challenges. In addition, there is a need to evaluate the implementation and knowledge dissemination efforts associated with mental health supportive aid programs such as mental health first aid [16].

Within the context of youth mental health, including psychiatric and psychological supportive programs, little is known about what knowledge translation (KT) strategies and theories were used to educate key stakeholders, including mental health practitioners when they transitioned to new models of care and implemented digital technology. Research is needed to evaluate what KT theories and models were used when disseminating knowledge to key stakeholders. Effective KT is a fundamental element of the public health research process [17]. Without effective translation of evidence into practice, research remains simply an academic area without real-world community health impact [18]. Understanding the key challenges and facilitators of KT, including implementing evidence-based mental health programs and interventions, is necessary. This way, future recommendations may be made for best practice guidelines during crisis times and for future pandemic preparedness. Given that there was an unprecedented rise in mental health issues during this time, it is important to know whether there were any KT strategies for parents, schools, and medical providers.

### Aims and Objectives

The purpose of this literature review was to gain deeper insights into strategies, programs, and services for child and youth mental health during the pandemic period. Recommendations for future program and intervention implementation, research, and best practice guidelines were made.

This review had the following aims:

1. To better understand the experiences, barriers, and facilitators regarding youth mental health service delivery, including telehealth, face-to-face, hybrid (combined face-to-face with digital), and school-based mental health service implementation, as well as psychological supportive services during the pandemic.
2. Secondary aims were to better understand what types of pediatric and youth mental health programs were implemented, including digital, face-to-face, and hybrid programs; the key stakeholders involved; and what KT theoretical models and strategies (if any) were applied during their implementation throughout the pandemic.

## Methods

### Overview

A literature review using PubMed and MEDLINE was undertaken to identify relevant studies on youth mental health programs and services, including school-based and hospital-based telehealth or hybrid implementation (combined in-person with digital services), and psychological supportive studies undertaken during the pandemic. Google Scholar and manual hand searches were also undertaken. We included studies that were undertaken during the pandemic period between March 2020 and October 2022. The studies must have mentioned that they were undertaken during the pandemic. The search was then updated and rerun with refined and more specific search terminology after consulting with a medical librarian to include studies that may have been undertaken during the pandemic but published at a later time up until December 31, 2023. Studies that were undertaken before the pandemic but continued throughout the pandemic were also included. The studies must have been published in the English language with public full-text accessibility. The rationale for including studies undertaken during the pandemic was to gain a greater understanding of key implementation challenges specifically during pandemic times of crisis and uncertainty. Google Scholar and manual hand searches were also undertaken to identify any additional studies. General studies on mental health service use or program trends without an assessment of experiences, preferences, and barriers were excluded. Psychological supportive studies were only included if they were part of a mental health service program or service implementation that assessed barriers, perceptions, and recommendations for implementation. Psychological studies that evaluated the effectiveness of the intervention on mental health outcomes rather than primarily focusing on stakeholder or user perspectives on the implementation of mental health services or overall telehealth experiences were excluded as they were outside the scope of this review. We included general positive psychology studies including mindfulness-based, cognitive behavioral, acceptance-based, emotional regulation, and behavioral activation strategies from stakeholder and user perspectives. Studies on the provision of mental health first aid training were only included if they evaluated implementation experiences or KT specifically. Studies that focused on framework development or development of implementation or KT models were included, but reviews or general opinion pieces were excluded.

The keywords included word variations of “knowledge translation” or “dissemination” or intervention and “mental health” or “resilience” or “stress” and “young adult” or “teenager” or “youth” or “child” and “health services” or “implementation” or “telehealth” or “psychological services,” among others.

An example of the search strategy is detailed in Textbox 1.

**PubMed search strategy example.**

**Search strategy**

- (“teen” [titleabstract] OR “teens” [title/abstract] OR “teenage*” [title/abstract] OR “adolescen*” [title/abstract] OR “youth” [title/abstract] OR “youths” [title/abstract] OR “young people” [title/abstract] OR “young adult” [title/abstract] OR “young adults” [title/abstract] OR “Child” [Medical Subject Heading (MeSH) term] OR “child*” [title/abstract] OR “student*” [title] OR “family” [MeSH term] OR “caregivers” [MeSH terms] OR “parent” [title] OR “parents” [title] OR “parental” [title] OR “familial” [title] OR “family” [title] OR “families” [title] OR “mother*” [title] OR “father*” [title] OR “caregiver” [title]) AND (“Depression” [MeSH term] OR “Depressive Disorder” [MeSH term] OR “Anxiety” [MeSH term] OR “Anxiety Disorders” [MeSH term] OR “stress, psychological” [MeSH term] OR “Mood Disorders” [MeSH term] OR “Depression” [title/abstract] OR “Depressive” [title/abstract] OR “melancholia*” [title/abstract] OR “suicide, attempted” [MeSH term] OR “Suicidal Ideation” [MeSH terms] OR “Suicidal Ideation” [title/abstract] OR “mental” [title] OR “psych*” [title]) AND (“mindfulness” [MeSH terms] OR “mindfulness” [title/abstract] OR “MBCT” [title/abstract] OR “MBSR” [title/abstract] OR “mindfulness based cognitive therapy” [title/abstract] OR “mindfulness based stress reduction” [title/abstract] OR “MBI” [title/abstract] OR “mindfulness-based interventions” [title/abstract] OR “meditation” [title/abstract] OR [“Mental Health”(MeSH term) AND “First Aid”(MeSH term)] OR “Psychological First Aid” [MeSH term] OR “mental health first aid” [title/abstract] OR “Psychological First Aid” [title/abstract] OR “resilience, psychological” [MeSH term] OR “resilience” [title/abstract] OR “hardiness” [Title] OR “posttraumatic growth” [title/abstract] OR “post-traumatic growth” [title/abstract] OR “personal growth” [title/abstract] OR “psychological well-being” [title/abstract] OR “stress related growth” [title/abstract] OR “coping behavior” [title/abstract] OR “emotional stress” [title/abstract] OR “flourishing” [title] OR “flourish” [title] OR “Emotions” [MeSH major topic] OR “positive psychology” [title/abstract] OR “Psychological Recovery” [title/abstract] OR “Mental Health Services” [MeSH terms] OR “psychological support” [title/abstract] OR “virtual” [title/abstract] OR “Teletherapy” [title/abstract] OR “eHealth” [title/abstract] OR “Telemedicine” [title/abstract] OR “telepsychiatry” [title/abstract] OR “cognitive behavioral therapy” [MeSH major topic] OR “mindfulness” [title/abstract] OR “meditation” [title/abstract] OR “mindfulness based stress reduction” [title/abstract] OR “MBSR” [title/abstract] OR “mindfulness based cognitive therapy” [title/abstract] OR “MBCT” [title/abstract] OR “acceptance-based” [title/abstract] OR “acceptance-based” [title/abstract] OR “acceptance and commitment” [title/abstract] OR “behavior therapy” [MeSH term] OR “behavioral activati*” [title/abstract] OR “behavioural activati*” [title/abstract] OR “activity scheduling” [title/abstract] OR “pleasant event*” [title/abstract] OR “pleasant activit*” [title/abstract] OR “daily diar*” [title/abstract] OR “behavioral therap*” [title/abstract] OR “behavioural therap*” [title/abstract]) AND (“diffusion of innovation”[MeSH term] OR “diffusion of innovat*” [title/abstract] OR “information dissemination”[MeSH terms] OR “knowledge util*” [title/abstract] OR “knowledge uptake” [title/abstract] OR “knowledge transfer*” [title/abstract] OR “knowledge implement*” [title/abstract] OR “knowledge disseminat*” [title/abstract] OR “knowledge translat*” [title/abstract] OR “research utiliz*” [title/abstract] OR “research uptake” [title/abstract] OR “research transfer*” [title/abstract] OR “research implement*” [title/abstract] OR “implementation” [title/abstract] OR “research disseminat*” [title/abstract] OR “research translat*” [title/abstract] OR “health services research” [title/abstract] OR “utili*” [title] OR “program*” [all fields] OR “school-based” [all fields] OR “implement*” [all fields] OR “train*” [all fields]) AND (“2019 NCOV” [title/abstract] OR “coronavirus” [MeSH term] OR “coronavirus” [title/abstract] OR “COV” [title/abstract] OR “COVID-19” [MeSH term] OR “COVID-19” [title] OR “COVID-19” [MeSH terms] OR “NCOV” [title/abstract] OR “Pandemics” [MeSH terms] OR “sars cov 2” [title/abstract] OR “sars cov 2” [MeSH term] OR “sars cov 2” [title/abstract] OR “severe acute respiratory syndrome coronavirus 2” [title/abstract]) AND (2020/01/01:3000/12/12 [publication date] AND “english” [language])

### Screening and Data Extraction

Titles were screened for relevance followed by screening of abstracts against the inclusion and exclusion criteria. The full texts of abstracts meeting the inclusion criteria were further screened. If the full-text articles met all the inclusion criteria, they were included in the literature review.

To ensure that the studies were undertaken during the pandemic period, we screened titles with the words “COVID-19” followed by checking the full texts to ensure that the programs were implemented within a COVID-19 context or with relevance to the pandemic, where lessons could be learned. Where it was unclear, authors of selected papers were contacted directly to confirm.

Data were extracted and summarized in tabular format. This included the study general characteristics, measures, outcomes (mental health, knowledge, and program implementation), KT media, and KT theories and behavior change theories.

## Results

**Figure 1:**
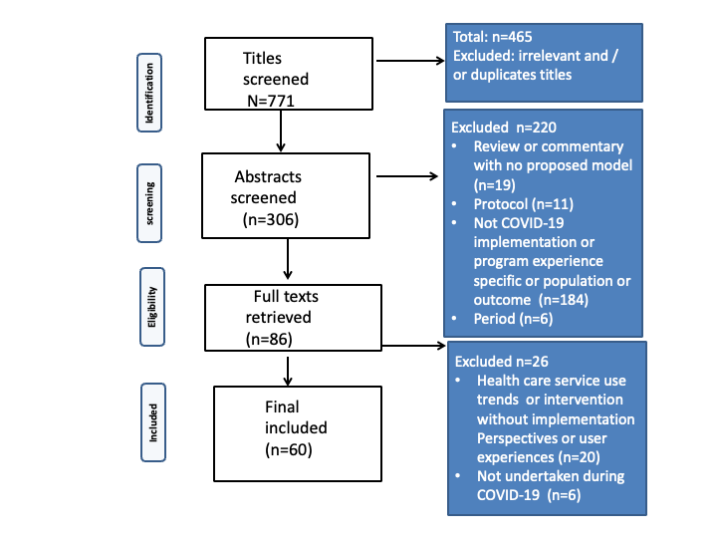
Flowchart.

A total of 60 studies on youth mental health service or program implementation were included in the final review [9,19-77]. Figure 1 illustrates the search and screening process.

### Mental Health Outreach Services and Programs for Children and Teenagers With Mental Health Problems

#### Youth Mental Health Service Implementation

We identified and included 60 studies on mental health program initiatives to promote child and youth mental health during the pandemic that focused on the delivery and implementation of a wide range of mental health services, programs, and supports as well as knowledge dissemination during the pandemic. The results are summarized in Table 1. The study types were qualitative studies, case studies, and cross-sectional studies, as well as studies with mixed methodology. The countries spanned Canada, the United States, and Australia. One large study was undertaken in Europe across 8 countries: Austria, Germany, the Netherlands, Slovenia, Switzerland, Italy, Sweden, and the United Kingdom [29].

**Table 1:** Child and youth mental health knowledge translation (KT) tools, strategies, and interventions among stakeholders.

Study, year	Location and design	Sample size, N	MH^a^ outcome and target group	Stakeholders	KT media—theory, BCT^b^, and diverse media?	KT dissemination plan or efforts or implementation plan?	Key findings and messages
Zbukvic et al [46], 2022	Australia; case study on workforce development KT	4400 MH workers	Teenagers with depression, anxiety, psychosis, trauma, borderline personality disorder, and self-harm (needs before and especially during the COVID-19 pandemic)	Work with stakeholders such as GPs^c^, psychiatrists, psychologists, social workers, peer support workers, teachers, the legal justice system, police, and addiction specialists	Training in person and web-based (also outreach visits to clinical practice areas to change practice based on best practice evidence); diverse media: fact sheets, videos, webinars, web-based modules, and games to enhance engagement; focused on sharing knowledge from research; used behavior change theory; used the i-PARIHS^d^ model (usability, context, innovation, facilitation, and recipients)	Orygen—youth MH organization focused on KT; primary, secondary, and tertiary health service delivery; offers professional development and continuing education support to enhance MH youth outreach	Difficulties with web-based e-MH care during the pandemic; levels of readiness across different organizations and adaptability; need for advice and recommendation and frameworks for implementation in a digital world using digital media
Parrot et al [37], 2022	Canada; focus groups and surveys	—^e^	Depression, anxiety, and germaphobia (pandemic related); kids (aged 6-16 years)	Work with key stakeholders at schools: parents (mean age 40 years; families), teachers, retired school board staff, retired teachers, and MH practitioners	Training in person and on the web on the DREAM^f^ program—founded on the Knowledge Transitional Integration Framework (4 pillars: sustainability, accessibility, credibility, and feasibility; developing a digital program and a social literacy program, and creating meaning through creative engagement [eg, music and arts]); online classes founded on positive concept; new learning mindset or beginner mind and gratitude; incorporated rational emotive behavioral therapy and rational emotional logotherapy (identifying emotions)	KT and dissemination; stakeholders actively engaged (addressed needs of stakeholders) and participated in the research process and in the KTA^g^ process—development, implementation, and evaluation stages; stakeholders were involved in surveys, focus groups, and interviews	Grounded theory informed; evaluated the credibility (hybrid program for those who do not agree with web-based methods), acceptability (stakeholders tried the web-based technology, and exposure increased positive perceptions; need for diverse music options with lyrics), accessibility, feasibility (ensuring extra teachers offer e-version in addition to the school-based version), and sustainability (memory aids, booklets, reminders, and program games based on key emergent themes from the interviews); recommendations for a hybrid in-person and web-based model for all students; the implementation stage of the aforementioned recommendations is still underway
Hou et al [30], 2022	Canada; cross-sectional survey	94; 84% female	Impact of COVID-19 on MH: depression, anxiety, and PTSD^h^; teenagers and young adults aged 13-39 years (cancer survivors)	Lived experience—childhood cancer survivors	Young adult cancer survivors had worse MH during the COVID-19 pandemic; infographic with information on meeting the needs of cancer survivors for MH during the COVID-19 pandemic (after a preliminary survey on MH); KT on coping strategies	Provided the infographic to those with lived experience with statistics on MH during the COVID-19 pandemic as well as coping strategies that were evidence based: increase sleep, improve diet and activity, and practice mindfulness	Infographic is just one medium; more research is needed on feasibility and the use of diverse media or resources for KT and dissemination; recommendation of personalized interventions with enhanced accessibility; more context research is needed (qualitative)
Hanson et al [29], 2022	Europe; mixed methods—country case studies, analyses, implementation of KT, and implementation of a prevention intervention (clinical trial in 6 countries in the EU^i^)	9437	AYC^j^ of family members aged 15-17 years; promote MH, well-being, and resilience	8000 stakeholders at national and international levels: health care professionals, schools, teachers, nurses, psychologists, politicians, social workers, and researchers across Austria, Germany, the Netherlands, Slovenia, Switzerland, Sweden, the United Kingdom, and Italy; expert panel on AYC	Intervention founded on ACT^k^ and the Discoverer, Noticer, Advisor model (derived from ACT); diverse media: in person, over Zoom, and using an app	ME to WE project involved several stages: knowledge synthesis via reviews, surveys of those with lived experience (needs), prevention intervention, qualitative interviews, and understanding of national policies and laws; participatory design with evaluation and impact of KT; KT and dissemination targeted stakeholders at meetings and networking efforts centered on raising awareness, engaging stakeholders, and involving them in the policy process	Key messages: identify young carers and offer supportive services, apps for support, peer and family support, and respite
Nicholas et al [35], 2021	Australia; cross-sectional survey	308 youths aged 12-25 years; 92 clinicians	General youth MH	Youth with lived experience and physicians	Telehealth implementation	Strategies to implement and improve the implementation of telehealth or tele-MH during the COVID-19 pandemic	Telehealth was not viewed as negative by young people in terms of attendance and perceptions of willingness to attend MH care relative to MH practitioners; technology issue barriers reported in 31% of cases; accessibility to e-MH and privacy were important
Eapen et al [25,78]	Australia; lexical analysis	6	Children with MH issues	Clinicians	e-MH service implementation	Strategies to ameliorate the implementation of e-MH by assessing the barriers and facilitators	Barriers to implementing e-MH included space, privacy, and technology (including internet accessibility); recommendations included a hybrid model with face-to-face interaction for real-life clinical assessment and web-based assessment to increase accessibility
Barney et al [20], 2020	United States; case analysis of the young adult medicine clinic telemedicine intervention implementation	—	General MH and addiction in teenagers and young adults	Clinicians (informing clinical practice) and MH practitioners (nurses, students, social workers, and administrative staff)	Telemedicine consultation implementation	Implementation of telemedicine	Increase in use by 97% during the COVID-19 pandemic; benefits were being feasible and acceptable, but barriers included privacy and technological barriers (eg, accessibility to devices); Zoom was preferred for privacy; need for clear guidelines for effective implementation
AlRasheed et al [19], 2022	United States; survey	285	Children and teenagers; depression, anxiety, trauma, and behavioral issues	Telehealth practitioners and children and youth with lived experience	Telehealth implementation	CBT^l^+psychology telehealth implementation barriers and acceptability	Difficulties interacting with children; privacy issues (teenagers less willing to speak about discrete matters at home); high acceptability among clinicians (78.2%)
Carretier et al [22], 2022	France; qualitative interviews	20	Depression, anxiety, and PTSD	Telehealth practitioners, parents, and youth with lived experience	Telehealth implementation	Tele-psychiatry implementation barriers and facilitators	Hybrid method recommended: face-to-face with web-based methods; practitioners felt that nonverbal communication could be a challenge; the quality of the therapeutic relationship influenced care and the severity of their patients’ MH problems; preference to select phone versus video calls; teenagers appreciated having their parents involved
Conradi et al [9], 2022	United States; case study implementation of telehealth at a children’s advocacy center	—	Children; general MH and trauma	Telehealth providers and children at a children’s advocacy center and their families	Informed by the Consolidated Framework for Implementation Research; online telehealth media	Implementation of telehealth; 3 areas: agency requirements, the needs of the telehealth professionals and training, and use of technology+effective communication strategies	Challenges included health professional burnout and need for support; need for clear communication and effective planning; patients should receive a virtual tour+“welcome kit”+frequent feedback on the sessions; ameliorate body language in front of technology; provide families with technology and adequate internet connection and play kits for the children; kids had “Zoom fatigue”; implementation of team managers for creativity+innovation; technology needs a consideration of space
Craig et al [23], 2021	United States; case study Affirm we-based group session	1 transgender youth	General MH in youth	Telehealth practitioners and LGBTQ^m^ youth social workers	Implementation of affective CBT; AFFIRM^n^ behavioral approaches; cognitive restructuring approaches; affirm social support networks; online and app support media	Evaluate the implementation of AFFIRM by engaging with LGBTQ youth	Recommendations: adapt to using Zoom (eg, chat and screen sharing) and become experienced with technology, include frequent check-ins and icebreaker questions to reduce virtual awkwardness, implement document sharing and Microsoft PowerPoint for engagement and dissemination, integrate calming strategies, and facilitate social networking via social apps for support
Doan et al [24], 2021	Canada; framework development proposal analysis of the 6 Pillars framework at the Hospital for Sick Children	—	General MH; children+teenagers	Clinicians, children, teenagers, and families	Implementation of virtual MH care for children	Implementation of the telehealth 6 Pillars framework at the Hospital for Sick Children, Toronto, Ontario, Canada; plan to do act assisted clinicians to come with guidelines for virtual care (2 weeks of meetings)	The physical space is important—private and clean for training and practice; providers should be accepting and ready to use technology; need for a safety plan in virtual meetings for emergencies or unexpected events; parents must be present during consultations; patients must find telehealth acceptable, and the environment must be suitable (eg, safe, technology, accessible, and private); patients need adequate physical space for privacy; clinicians must follow and adhere to clinical practice standards and laws during telehealth
Gorny et al [27], 2021	United Kingdom; case analysis of London ward	36 hospitalized during the first-wave lockdown in March 2020	General MH problems, depression, and anxiety	Physicians, politicians, the national health care regulator (Care Quality Commission), hospital staff, social workers, children, parents, and leadership representatives from MH trusts	Implementation of a child and youth MH ward; —	Ward was created for child and youth MH to meet the COVID-19 challenges with a change in organizational structure and leadership; multidisciplinary team morning meetings to plan and work on patient cases	Challenges with implementing a young people and children MH ward in a pediatric setting; plan to set up a new ward involved integrating physical+MH care; MH nurses and child psychiatrists were allocated to one place in proximity to patients; dynamics changed with a multidisciplinary team and new managerial or leadership approaches for managing MH; tailoring treatment to patient needs
Lal et al [32], 2022	Canada; cross-sectional	51	General MH	Young adults	Telehealth implementation	Assess implementation barriers and experiences with youth telehealth	Main barriers included technological (technological support needed for the preliminary sessions) and privacy (secure private connections) barriers; overall, those with lived experience or knowledge users found the telehealth platform to be acceptable
Moorman [34], 2022	United States; analysis of practice data	100; 40 clinicians and 60 families	General MH	Practitioners, children and teenagers, and parents	Telehealth implementation (digital media)	Assessment of implementation challenges and experiences	Benefits of telehealth included higher attendance and access to MH; challenges with younger kids; difficult to understand body language web-based (nonverbal communication); privacy concerns
Palinkas et al [36], 2021	United States; qualitative interviews via Zoom	21 state MH authorities	Range of MH problems in children+teenagers	State MH authorities	MH care provision, including telehealth	Rapid assessment procedure used to analyze key implementation themes regarding youth MH service delivery	Barriers to telehealth: technology+internet, privacy issues, challenges with using telehealth with younger children, and need for greater training of health professionals and an integrated system of reimbursement of costs of treatment
Power et al [39], 2022	Ireland; toolkit design	—	General MH promotion via+MH	Children and primary and secondary MH practitioners	Print resources (leaflet) for KT for promotion+MH; coping strategies; mindfulness; positive psychology; hand model of the brain	KT; happiness toolkit of essential MH resources created; KT of 6 evidence-based techniques for MH and resilience building; kids had to build an actual box; includes advice on social support or building healthy relationships; mindfulness; positive visualization—smile a vision; gratitude; hugs; hand model of the brain to identify emotions	Focused on the development of a model for MH care; the MH needs of children (pediatric MH population) are not met when physicians are not adequately trained; the toolkit may enhance mental well-being as a model of MH care
Randall et al [40], 2021	Australia; mixed methods—health data sets and surveys	140	General MH	Children and teenagers with MH problems and MH clinicians	Digital telehealth (psychiatry)	Challenges and opportunities in the implementation of telehealth	Barriers: technological issues identified as barriers, lack of human contact, and younger kids preferred face-to-face over video consultations; benefits were convenience; provider challenges included adequate training—developing skills for interviewing
Sharma et al [42], 2020	United States	57 faculty members and 97 clinicians in need of training	General MH in children and teenagers	MH clinicians at Seattle Children’s Hospital: nurses, psychologists, psychiatrists, and behavioral therapists	Telehealth; tele–group training	Tele-psychiatry implementation involved phone consultations as a bridge before the full transition; took 6 weeks to implement tele–group training for MH practitioners; TeleMed Home scheduling visits and administrative staff trained	Clinicians have limited training in tele-psychiatry; tele-MH has barriers: need for special training for the DTC^o^ virtual platform; privacy issues regarding using third-party applications for consultations; technological issues with the DTC platform crashing
Skar et al [43], 2022	Norway; clinical treatment data analysis	382 therapists treating 1240 patients	Children and teenagers with trauma	MH practitioners; 66 clinics	Trauma-focused CBT implementation	Issues with the implementation of CBT	Adequately trained (high intense) therapists linked with patient response and continued attendance; leadership training also important (secondary) to intense training for higher fidelity and nondropout; nonresponse linked to older children
Somaiya et al [44], 2022	Australia; qualitative focus groups	44	General MH; youth	Youth with MH problems	Digital telehealth	Experiences with implementation of telehealth	Successful implementation of telehealth, acceptable+feasible; preference for face-to-face as an option after the pandemic in youth; benefits included accessibility, convenience, and web-based social interaction; barriers with technology, internet issues, lack of human contact and privacy, space, comfort with being on the internet, and meeting time or scheduling problems
McMellon and MacLachlan [33], 2021	Scotland; case or policy analysis	Aged 10-18 years	General MH	Observatory of Children’s Human Rights in Scotland, Children and Young People’s Commissioner Scotland, and children	Impact analysis of MH legislation for children during the COVID-19 pandemic	Recommendations by the ICRA^p^ made for KT and implementation for the government	Increase resources for children’s MH, including in schools (eg, training more staff to meet capacity and enhance access or reduce barriers to e-MH via technology and internet access for kids); offer in-person support for special groups who need it; undertake more research on special needs in diverse groups
Zickafoose et al [47], 2022	United States	29,000 surveys; 400 randomly selected who undertook trauma-informed training interventions	Children and teenagers; MH trauma	MH America schools and MH professionals	Dissemination and training for trauma-informed classroom practices	Implementing informal MH training and help via schools combined with formal professional medical help	Trauma-informed classroom practices can be implemented with effective dissemination via professionals and school staff; there were no differences in knowledge acquired when school staff versus health professionals delivered the MH material in trauma-informed classroom discussions; requirements include quality training
Rodriguez-Quintana et al [41], 2021	United States	982 trainees for the session; 155 needs assessment surveys	Ages from kindergarten to 12th grade; general MH	TRAILS^q^ program	Group manual for school-based health professionals for youth MH; coping skills; promotes CBT+mindfulness (includes emotion recognition and gratitude); implementation of TRAILS program in schools; digital media: virtual support and information or tools; informed by therapeutic principles	Handbook or manual for knowledge dissemination and training for youth MH website with materials or information; 3-hour virtual training	The study focused on the development and early implementation; overall satisfaction among health professionals with the training materials; overall acceptability; some providers reached out for support with using virtual meetings and interactive web-based material; lack of internet may be a barrier for students and providers
Podar et al [38], 2022	Germany; mixed methods—semistructured qualitative interviews+longitudinal data	12 gatekeepers and 216 adolescents	General MH; teenagers (refugees and migrants)	YOURCARE research project participants; schools+teachers, teenagers, parents, and psychologists	Needs assessment and case analysis of COVID-19 MH; media: —	Implementation and dissemination needs and barriers	Lack of integrated MH care in schools; segregation and stigma among young refugees; barriers to accessing MH care; need for antidiscrimination policies in schools; COVID-19 perpetuated inequalities; diversity is needed in implementation and research by including migrants
Weineland et al [45], 2020	Sweden; qualitative semistructured interviews	14	General MH in youth	Primary care therapists; youth MH centers in Sweden	Internet implementation of CBT	Implementation experiences with iCBT^r^ training and program delivery	Barriers included motivating patients and the therapeutic relationship when communication is not in person
Goddard et al [26], 2020	United States; qualitative case analysis	308	General MH in youth	Listening and Learning attendees from the School-Based Health Alliance; school-based health centers	KT strategies to adjust to the pandemic on the Listening and Learning platform; digital implementation of telehealth (general); MH as a topic; digital media: Zoom, Skype, and online platform	Listening and Learning KT platform developed to help providers during the COVID-19 pandemic; experiences with implementing telehealth and strategies; physical art supplies and kits for children for MH (eg, dialectical therapy mailed to their homes)	Most providers adapted to virtual MH care using Zoom, Skype, FaceTime, Google Classroom and Voice, and Doxy; most barriers were technological issues such as troubleshooting; benefits included greater parental involvement; concerns about the privacy of the teenagers
Khan et al [31], 2021	United States; development of training and dissemination	—	General MH in children and teenagers	American Academy of Child and Adolescent Psychiatry, American Psychiatric Association, and directors of psychiatric training websites	6-stage model of curriculum development by Kern	Web-based curriculum for KT on pediatric tele-psychiatry; implementation addressing patient needs; 6 areas of competency; resources; evaluation tools for practitioners	Currently available on the internet for training; further results will come
Birkenstock et al [21], 2022	United States; virtual MH workshop implementation, community-based participatory research study	11	Immigrant youth with MH problems	Refugee immigrant center; youth with lived experience; university+art and health collective; Sanctuary Philadelphia Independent Cultural Youth project	Art workshops for MH; developing strategies for youth MH based on needs or planning	Implementation of an MH project for youth migrants on the web from face-to-face due to COVID-19	Issues with implementation due to COVID-19; challenges with switching to a digital system; fatigue from web-based meetings; more complex artwork was not possible on the web; alternative expression included photos, videos, and memes; activities mainly centered on virtual discussions; care packages sent to participants; program terminated early due to lack of participation
Haliwa et al [28], 2022	United States; qualitative	191	General MH in school-age youth	—	Mindfulness on the web	Kidding around; yoga; implementation of a school-based digital training of teachers mindfulness program; knowledge, attitudes, and perceptions regarding implementation of mindfulness in schools	Misperceptions of mindfulness may act as barriers to implementing it in schools; cultural acceptance issues potentially; benefits for emotions and coping skills
Danseco et al [54], 2021	Canada; multilevel mixed methods implementation research—surveys+focus groups	192 youth and 97 agency leaders (surveys only)	General youth MH (virtual)	Families and youth, agency leaders, and MH service providers	Virtual care platform	Implementation of a virtual care platform for youth; adapted the Consolidated Framework for Implementation Research	Common barriers to implementation: privacy, internet connection, resources (eg, laptops and phones), and safety (sudden disconnection and unknown risk to the patient or a connection issue); less engaging than face-to-face for some (eg, children)+nonverbal cues harder to assess than face-to-face; work-life balance between home and the office and loneliness for MH practitioners; facilitators: engaged with adequate training and leadership as well as collaboration and good relationships with patients
Davenport et al [55], 2020	Australia; proposal for a model of care^h^	—	General MH	Tailored to MH for youth	Web-delivered care; digital toolkit	Brain and Mind Centre model of care; health IT for model deliverance; web-delivered care; Innowell platform	Need to implement necessary technological infrastructure for this model of care (implementation experiences not described)
Dhonju et al [57], 2020	Nepal; implementation of a multitier platform	100 health care professionals; 1206 parents, teachers, and caregivers	General MH	MH care professionals, teachers, and parents	Web-based platform with the use of apps for training, such as Zoom, Microsoft Teams, and Google Hangouts	Implementation of an MH platform or telehealth with face-to-face in-person programs	Challenges with participation (finding MH professionals to engage with); remote issues; connection issues with the internet and resources (access to technological devices); challenges with learning how to use Zoom for de novo users; barriers with timing and scheduling virtual sessions; resource issues: not all remote schools have access to the technology for virtual sessions and fewer interactional possibilities on the web versus in person; stigma is also a barrier
Havewala et al [75], 2023	United States	35	MH first aid training	Youth	Digital MH training	MH first aid training implemented virtually	Improvements in MH literacy, knowledge, confidence in MH first aid, help-seeking attitudes, and stigma
Hawke et al [61], 2021	Canada; survey+open-ended questions	491	General MH in youth	Youth with and without lived MH experience	Explored use of virtual care, including web-based video sessions, phone calls, and use of messages	Not a specific implementation study but one on general experiences with accessing virtual care	Youth preferred MH virtual services that involved web-based video sessions, followed by phone calls; messages were the least preferred; technology had to run smoothly, including preferences for quality internet connection and technology; ease of booking and free MH care preferred; privacy and ability to mute were important; desired content on mindfulness and fitness and engaging content with education; preference for one-on-one over group sessions; need for human personal connection
Markoulakis et al [65], 2022	Canada; qualitative study, semistructured interviews	46	General MH	Youth and caregivers (from the standpoint of carers)	General virtual care and face-to-face services	Not implementation specific but about experiences with MH services, including digital ones, in Ontario, Canada	Fragmented MH services with difficulty accessing them; virtual care benefits included accessibility without the need to travel; issues with engagement with virtual (being easily distracted and it feeling less personal) versus in-person care; barriers with technology issues, including Wi-Fi
McQueen et al [66], 2022	Australia; qualitative (3 participant groups)	167 health care professionals and 68 parents	General MH	Parents and MH care providers	General MH services and virtual care	Not implementation specific but about experiences with MH services	Telehealth barriers included privacy; technology issues with Wi-Fi, sound, and camera (image quality); benefits of telehealth included accessibility; preferences for a hybrid on the web+in-person approach
Meininger et al [77], 2021	Germany; survey	561 therapists and 227 parents	General MH	Parents and MH professionals	Telehealth	Not implementation specific; general experiences with MH services and telehealth at an outpatient unit	One-quarter transitioned from in-person care to telehealth; high satisfaction+acceptance of telehealth; 47% of therapists preferred in-person care over telehealth (over returning to the latter); technology requirements were a barrier in 19% of cases (did not undertake telehealth)
Purtle et al [68], 2022	United States; surveys	159 state officials	General MH	State officials	General MH services+telehealth	Not implementation specific; general experiences with nationwide MH services+telehealth	Main barriers to telehealth were due to remote location, access to technology, and internet connection
Rudnik et al [69], 2021	Poland	—	General MH	Students	Psychological support delivered via email+Skype; video calls; self-care behaviors; relaxation	Academic Psychological Support Centre in Gdańsk, Poland; applied the pandemic management theory; psychoeducation	Proposed model of care; initial challenges concerned a lack of clear guidelines
Brahmbhatt et al [49], 2021	United States and Canada	22 hospitals	General pediatric MH	Hospitals and MH practitioners	Tele-psychiatry; use of Zoom, WebEx, FaceTime, Microsoft Teams, Epic, Google, phone calls, video consultations, and a combination of media	Tele-psychiatry implementation	Rapid transition to tele-psychiatry used in 16/22 practices (PCLPs^s^); challenges with resources, including access to technology in 75% of cases; access to essential technology rose later on in the pandemic; quality of care not a challenge during virtual as well as hybrid delivery of MH services; lower levels of preference for virtual MH care among primary care providers; challenges faced due to a lack of continuity between primary care physicians and psychiatry in their use of tele-psychiatry
Campbell et al [50], 2023	Canada	48 for the semistructured interviews and 1300 for the mixed methods survey	General MH	MH practitioners, caregivers, and patients	Telehealth	General telehealth implementation experiences	Telehealth is helpful, but hybrid models are preferred; there was diversity in preferences; preference for in-person care for stronger therapy cases; need for private, safe space for consultations and access to technology; usefulness of telehealth included accessibility and no need to travel+social distancing; personal factors need to be considered when deciding the treatment modality and setting; facilitation of telehealth enabled through technological assistance guided by MH care providers
Campos-Castillo and Laestadius [51], 2022	United States; cross-sectional	532	General MH; teenagers (AmeriSpeak Teen panel)	Teenagers	Telehealth and chat-based messages	Experiences with using the MH care system; not specific to one implemented program	Support from the teenagers’ parents critical for enabling access to telehealth, including the provision of space; barriers to equitable access to in-person care among Black teenagers with preferences for in-person support; greater use of messaging and chat-based approaches for ethnic minority groups; need to ensure racial equity in MH diverse digital service accessibility
Childs et al [53], 2020	United States; observational implementation study at a psychiatric hospital	—	General MH	MH care providers at a psychiatric hospital managing teenage patients with MH problems	Zoom, Epic, video group-based psychotherapy, and Telephonic	Telehealth, including Telephonic, MyChart, and video consultation; implementation of youth psychotherapy at the hospital	Telehealth is feasible for group-based psychotherapy; need for an integration of telehealth with the electronic medical record for long-term sustenance
DeJong et al [56], 2022	United States; cross-sectional survey	138	General MH	Psychiatrists	Telehealth	Evaluation of telehealth training, education, and care	Large uptake of telehealth linked to a greater ability to manage the technology; fewer barriers in clinical care training using telehealth reported and greater training relative to prepandemic levels; barriers that remain need for greater equity in telehealth accessibility and funding
Garbutt et al [59], 2022	United States; qualitative interviews	19 pediatricians and 2 nurses	General MH	Pediatricians and nurses	—; provision of general MH support	Experiences with providing MH services; the Child Psychiatry Access Project	Discontinuity between care provided by pediatricians and MH specialists; need for greater accessibility to advice from colleagues on integrating MH care in the home setting; need for comprehensive MH care implementation for remote patients
Gorfinkel et al [60], 2023	Canada; cross-sectional survey	1928	General MH	Teenagers	Video, phone calls, chat-based support, and in-person support	Perspectives and experiences with receiving MH services	Preference for in-person MH care, followed by SMS text messages; phone calls were less preferred, second to video tele-MH consultations
Lal et al [63], 2023	Canada; longitudinal survey	26	General MH	MH care providers: clinicians, nurses, social workers, occupational therapists, and peer support	First-episode psychosis telehealth platform	Tele-psychiatry perspectives using the Reacts platform	Preliminary barriers that subsided with use included technology issues: sound, image quality, and internet connectivity; benefits: patient engagement+accessibility to continued MH care; benefits of Reacts: user centered and private
Malik et al [64], 2023	India; mixed methods—survey+qualitative interviews	34	General MH	Teenagers and counselors	Digital behavioral problem-solving; tailored behavioral model; voice and video calls	Remote stepped-care model of mental health	Teenagers: preference for voice calls, which felt more private and required less technology; counselor barriers: timely remote support and need for teamwork from both the patient and provider to meet specific needs
Olson et al [67], 2020	—	—	Suicide prevention training	Families+caregivers	Zoom; Northwest Mental Health Technology Transfer Center	Involved patients with their families in the process; Northwest Interconnected Systems Framework; outreach efforts informed by Consolidated Framework for Implementation Research	Switching from in-person to web-based suicide prevention training resulted in greater participation levels than in prepandemic times; increases in knowledge+changes in behavior
Rusu et al [70], 2023	The Netherlands	1065	Cross-sectional survey on experiences	Psychiatrists	Telehealth	—; general experiences with telehealth	The stakeholders were more accepting of telehealth if they had previous training and clinical experience; recommendations to provide both for its successful implementation
Schriger et al [71], 2022	United States; mixed methods—survey and interviews	45	General MH	MH clinicians	Telehealth	—; general telehealth experiences	Training is essential for telehealth; need for resources; engagement is impacted during telehealth+discussion focus or topics; telehealth is not uniformly great for everyone and is contingent on patient characteristics and preferences; higher level of involvement, engagement, and creativity in some patients and families in virtual settings; barriers: privacy and internet accessibility
Stuart et al [72], 2023	Canada; cross-sectional survey+focus groups	29	General MH	ED^t^ physicians	Telehealth	—; general telehealth experiences	Barriers to finding the time for telehealth and lack of resources or support from others; need for a greater level of training and comprehension on specific duties and roles in telehealth in the ED setting; benefits: value in providing services virtually for assessing MH
Sullivan et al [73], 2022	Semistructured interviews	207 (121 school-based health centers)	General MH	School-based centers	Zoom; School-Based Health Alliance (60-min session)	—; discussion over Zoom about school-based mental health support recommendations	Key barriers included limited availability of in-person support and staffing; community-based partnerships were important for the sustained provision of support; other barriers were language, cultural, privacy, and technology related (internet and Wi-Fi)
Williams et al [74], 2023	United States; pre- and posttest analysis of the electronic health record	—	General MH	Pediatric MH care providers at the hospitals	Telehealth	—; assessment of the electronic health record	Barriers and accessibility issues for pediatric telehealth in patients from racial minority groups
Kaar et al [62], 2023	Single-arm pilot study that transitioned during the pandemic from Colorado to Alabama (Ally)	—	General MH	Schools, students, and MH volunteers	Web-based delivery of Ally (advocates for all youth)	Health equity implementation framework—FRAME; use of volunteers	Adaptation to the new community setting and implementation required greater community support with volunteers to drive the program; 30-minute sessions each week; challenges with maintaining an equity perspective and health literacy level diversity; need for representation in group leaders from the same backgrounds; the benefit of virtual delivery was a greater reach
Banks [48], 2022	United States; open-ended survey	33	General MH	Teenagers	—	—; general health care use experiences	Financial problems were a main barrier for Black teenagers when it came to accessing MH services
Childs et al [52], 2021	United States; pretest-posttest analysis of attendance rates during the pandemic	—	General MH	Youth and adults (only youth information included)	Telehealth compared with in-person care	Telehealth implementation	Telehealth helped increase accessibility; benefits for group therapy during the pandemic; racial inequities in accessibility to telehealth in Hispanic and Latino youth
Bhat [76], 2021	United States; survey	6393	MH in patients with ASD^u^	Families	Web-based MH services	Experiences with web-based MH services for children with ASD	Web-based MH was not always suitable for many families with children with ASD; recommendations for hybrid (in-person and on the web) MH care
Endale & Birm an et al [58], 2020	United States Case study commentary on program experiences	N/A	General mental health	Key stakeholders at the Kovier Centre Child Trauma Program	Transition to an online delivery of care with video calls outreach	Psychological first aid training Knowledge dissemination to families Translation to various language over text messages sent to families School coordination planning	Digital literacy barriers Access to technology and internet barriers Privacy barriers (space)

^a^MH: mental health.

^b^BCT: behavior change technique.

^c^GP: general practitioner.

^d^i-PARHIS: Integrated Promoting Action on Research Implementation in Health Services.

^e^Not applicable.

^f^DREAM: Developing Resilience Through Emotions, Attitudes, and meaning.

^g^KTA: Knowledge to Action.

^h^PTSD: posttraumatic stress disorder.

^i^EU: European Union.

^j^AYC: adolescent young carers.

^k^ACT: acceptance and commitment therapy.

^l^CBT: cognitive behavioral therapy.

^m^LGBTQ: lesbian, gay, bisexual, transgender, and queer.

^n^AFFIRM: Manualised Affirmative Cognitive Behavioral Therapy.

^o^DTC: Direct to Consumer.

^p^ICRA: Independent Children’s Rights Impact Assessment.

^q^TRAILS: Transforming Research Into Action to Improve Lives of Students.

^r^iCBT: internet-based cognitive behavioral therapy.

^s^PCLP: pediatric liaison psychiatric consultation provider.

^t^ED: emergency department.

^u^ASD: autism spectrum disorder.

#### Stakeholders

Most programs engaged stakeholders, including politicians, not-for-profit organizations, schools, teachers, parents, youth with lived experience, health professionals including general practitioners, psychiatrists, psychologists, social workers, and the criminal justice system [20,25,29,30,35,37,46,79]. A study in Europe used the Knowledge to Action framework [80], with stakeholders involved in each stage of the research process from the development and implementation stages to the evaluation stage, including focus groups, surveys, and interviews [37].

#### COVID-19 Context

Most of the articles explicitly described that the study was implemented during the COVID-19 pandemic in their titles [9,19-22,24,26-28,30,32-42,44]. Others discussed their study relevance in relation to the COVID-19 pandemic in the abstract or main text [9,23,29,31,38,39]. Several articles described that they were undertaken during the COVID-19 pandemic in both the abstract and title as well as in the main text [48-74]. A few were undertaken before the pandemic but continued throughout the pandemic and had to adjust their implementation strategies, including transitioning from face-to-face programs to adapting to digital health or COVID-19–specific needs [43,45-47].

#### KT Media

Several but not all the programs delivered mental health KT interventions through diverse media to their various stakeholders [23,26,29,37,39,41,46]. Diverse media included combinations of fact sheets, webinars, Zoom meetings, videos, modules, infographics, and toolkits. For example, Orygen in Australia provided primary, secondary, and tertiary mental health services and education to mental health professionals both on the web and in person along with outreach visits to patients. Their KT media included fact sheets, webinars, videos, web-based modules, and games to increase engagement. Another large study across Europe integrated face-to-face KT using digital tools, including Zoom and a KT app [29]. Several studies used a hybrid method involving face-to-face and digital KT media [22,25,29,30,33,37,46]. However, most focused on implementing e–mental health programs exclusively on the web, usually through virtual mental health or telehealth [9,19,20,22,24,25,28,32,34-36,42,44]. One study focused on using 1 KT medium for translating information on self-care during the pandemic to young cancer survivors to promote their mental well-being using an infographic [30].

#### KT Theories

A few of the programs applied specific KT theoretical models and frameworks that informed their program implementation [9,31,37,46]. For example, the study by Zbukvic et al [46] in Australia used the Integrated Promoting Action on Research Implementation in Health Services model. The model is founded on usability, the context of the KT intervention, facilitation, and the recipients [46]. In addition, the DREAM KT program in Canada adopted the Knowledge Transitional Integration Framework. The framework is based on 4 pillars, namely, sustainability, credibility, accessibility, and feasibility.

In addition to established KT models, a few positive health psychology theories or models derived from this field were used [23,28,29,37,39,41,43]. For example, a large study across Europe by Hanson et al [29] was informed by positive psychology using acceptance and commitment therapy that informed the Discoverer, Noticer, Advisor model. The study by Zbukvic et al [46] in Australia also integrated the behavior change theory model into the KT program. The program in Canada also focused on developing resilience through emotions, attitudes, and meaning in their youth KT mental health program, whereby they sought to maximize positive experiences in youth with a beginner’s mind and creative expression using means such as art, music, and gratitude [37]. They also integrated logotherapy through emotion identification into their program. Grounded theory further informed the first stage of their research at the stakeholder interview stage [37].

### Barriers to and Facilitators of Digital and Face-to-Face Mental Health Service Program Delivery and Implementation for Youth During the Pandemic

#### Digital and Tele–Mental Health Implementation

Several studies reported barriers associated with technology and privacy [19,20,24,25,32,34-36,42,54,61,66,72,73]. Zoom was preferred for privacy reasons when conducting tele–mental health consultations in one study [20]. Accessibility issues included access to technological devices and adequate internet connection [9,25,33,36,54,57,61,63,65,66,68,72-74]. The studies recommended that parents should have adequate training and an orientation on the technology as well as increased accessibility to the internet. Technological support was also needed for the preliminary tele–mental health sessions [32], and technological issues were identified in 31% of users in one study [35].

Space was identified as a critical feature for the successful implementation and delivery of virtual mental health services and programs to children and teenagers [9,24,25,50,51]. For example, the study by Doan et al [24] at the Hospital for Sick Children in Canada, which adopted the 6 Pillars framework in their development of a framework for mental health practitioners, emphasized that the space needed to be clean and private for medical providers.

Body language or nonverbal communication cues were also identified as barriers in some studies [9,22,34,45]. One study found that it was difficult to motivate patients and that work was needed to build the therapeutic relationship when mental health consultations were virtual as opposed to face-to-face [45]. In addition to body language, there were challenges with implementing certain digital mental health interventions, including art therapy programs on the internet during the pandemic [21].

A few studies also found that tele–mental health service implementation was more challenging in younger children [9,19,22,34,36,40,54]. Children preferred face-to-face mental health consultations over virtual ones [40]. In addition, one qualitative study in France by Carretier et al [22] found that teenagers preferred phone calls over video consultations. “Zoom fatigue,” feeling tired from using web-based technology for mental health care, was identified as an obstacle in one study in children. In total, 3% (2/59) of the studies made play or art kits for children to keep them engaged. One study recommended offering “play kits” to keep children interested and engaged with the technology [9]. Similarly, “art kits” were mailed out to children undergoing mental health therapy, including dialectical behavior therapy in adjunct to virtual telehealth appointments [26]. Thus, it seems that sending hands-on engaging resources to children may assist with their participation during web-based mental health sessions.

There were also challenges with managing and adapting to using the digital technology. For example, the case study by Zbukvic et al [46] on KT in workforce development (Orygen) involving 4400 mental health workers found that it was challenging to deliver digital mental health care during the pandemic and that there is a need for a framework with clear guidance on how to best deliver e–mental health care using digital media. They found that levels of readiness and adaptability varied across different stakeholder organizations [46]. Physician-level factors included the need for adequate training in tele–mental health [36,40,42,43] as well as readiness to partake in it [24].

A few studies found that physicians needed to be sufficiently trained in the technology when dealing with young patients with mental health problems. The study by Skar et al [43] found that there was higher patient attendance when they had adequately trained physicians. For example, this included leadership training [43] and learning how to undertake virtual interviews [40].

Actual hands-on training in using tele-psychiatry technology and the virtual platform was also emphasized in one study [42]. The study by Doan et al [24] also found that there is a need for a safety plan, with adequate preparation during emergencies or unforeseen events. The qualitative focus group study by Parrot et al [37] found that stakeholders who were not previously supportive of web-based mental health platforms found them to be acceptable after they had tried them, indicating that perceptions of digital mental health are more positive and willingness to try it increases if users learn how to apply it. The qualitative study by Goddard et al [26] involving the “Listening and Learning” platform for school alliances found that most providers adapted to using telehealth and virtual meetings on Zoom, Skype, and FaceTime. The study by Craig et al [23] found that there is a need for medical providers to be familiar with navigating digital technology, including screen sharing, implementing Microsoft PowerPoint for greater engagement and knowledge dissemination, and learning how to break social awkwardness on the web through things such as “icebreaker questions” when implementing cognitive behavioral therapy. They also found that there is a need to implement social apps for social networking [23]. Therefore, adequate knowledge dissemination on the use of digital health technology and its successful implementation through gaining interviewing skills and providing sufficient engagement for children and youth across diverse service providers is needed.

Overall, most of the studies that assessed acceptability and feasibility found that digital health and tele–mental health were viewed positively and there was a high uptake by youth during the pandemic [20,24,32,34,41,77]. The cross-sectional survey involving a needs assessment from the Transforming Research Into Action to Improve the Lives of Students implementation study (N=982 school-based health professionals who were trained) found that school-based mental health professionals were satisfied with the web-based manual that disseminated knowledge on youth coping skills [41]. Only one study found that medical providers experienced burnout from implementing telehealth [9]. The study by Moorman [34] that analyzed practice data from 40 health professionals and 60 families found that tele–mental health increased accessibility to mental health care for children and teenagers and resulted in a higher attendance.

#### School-Based and Nondigital Mental Health Approaches

A couple of studies examined the challenges with implementing mental health programs in face-to-face settings such as in schools and clinics. A large study in the United States with 29,000 stakeholders that analyzed data from 400 participants who undertook training in trauma-informed practices found that trauma-informed classroom practices could be successfully implemented through joint efforts between school-based professionals and mental health care providers [47]. The mixed methods qualitative interview study with 12 gatekeepers (with longitudinal data from 216 teenagers) by Podar et al [38] in Germany found that there is a need for an integrated mental health care system in schools and that inequities in accessibility exist among young refugees who experience stigma. They emphasized that there should be strong antidiscrimination policies when it comes to youth mental health in schools [38]. Thus, implementing programs in schools should maximize accessibility for all students. A case study analysis in the United Kingdom of youth in wards (N=36) found that it was challenging to implement a youth mental health ward in a pediatric setting, which required setting up a new ward and combining physical health with mental health [27]. They also found that it required joint efforts in a multidisciplinary team setting involving nurses and pediatric psychiatrists, which led to adopting new leadership and managerial approaches as well as tailoring treatments to individual patients [27].

### Hybrid Mental Health Approaches

Some of the studies opted for hybrid approaches when implementing mental health services and programs for children and teenagers [22,25,29,30,33,37,46,76]. The case analysis by McMellon and MacLachlan [33] in Scotland recommended a hybrid program with enhanced access to digital mental health services and technology in schools combined with face-to-face in-person support through greater staff training. The lexical analysis study by Eapen et al [25] in Australia also made recommendations for a hybrid mental health care service delivery for youth in clinics and at home to increase accessibility, ensuring clinical assessment in person combined with web-based support. Another study in Australia (case study) by Zbukvic et al [46] found that diverse stakeholders, including mental health care providers, required web-based training and outreach in-person clinic visits, with KT and dissemination strategies involving videos, fact sheets, modules, and webinars to meet the mental health needs of youths. In addition, a large mental health mixed methods KT implementation study in Europe (ME to WE) involving 8000 stakeholders evaluated KT and dissemination strategies to meet the needs of youth who cared for someone [29]. Through extensive knowledge syntheses and a participatory design, they found that youth should receive support from both peers and family in addition to mental health supportive services and respite. They also made recommendations for a mental health app that would support young caregivers [29]. Raising awareness of the mental health needs of young carers was also brought up as a theme [29]. The focus group study by Parrot et al [37] recommended a hybrid model for in-class and web-based KT for youth mental health by ensuring that teachers offered both the in-class learning version and the web-based one. Behavior change techniques such as reminders to enhance memory were also recommended, including things such as games, booklets, and memory aids. They also found that students preferred a range of lyrics with music as part of a meaningful program [37]. To enhance accessibility and engagement, they also recommended more teachers who could offer the program on the web [37]. The study by Hou et al [30] found that an infographic, while informative, was only one medium for KT and dissemination for youth mental health and resilience and that other KT media should be explored in the future along with personally tailored interventions.

In addition, one study combined passive KT with active KT, a type of hybrid approach for disseminating knowledge. The study by Power et al [39] involved a KT leaflet for resilience building in children and teenagers combined with a happiness toolkit that required children to build a physical box, emphasizing the importance of real-life relevance for children in addition to a passive leaflet when it comes to participation and engagement. However, this was an early developmental study on a new model of care rather than an implementation study evaluating its barriers and acceptability.

Overall, support services should include a range of resources, including in-person social support and an app. All these studies with hybrid approaches found that accessibility to mental health supportive services or engagement was enhanced when diverse options were offered through in-person support, web-based support, digital applications, and support from family and friends in addition to professional help to accommodate diverse learning needs and preferences. As barriers were noted for digital delivery, hybrid methods were also recommended for accessibility from this perspective. It also appears that active versus passive methods may enhance participatory engagement; however, more research is needed to confirm these findings.

### Mental Health First Aid

A couple of studies implemented mental health first aid through virtual platforms and found that knowledge improved [67,75]. The study by Olson et al [67] found that participation in suicide prevention training increased when they transitioned to a web-based delivery system due to COVID-19.

### Summary of Findings

In summary, it seems that the successful implementation of mental health programs requires hybrid approaches involving both in-person (face-to-face) and web-based sessions. Digital mental health may be successfully implemented if barriers are minimized, such as providing children, teenagers, and their families with adequate access to the internet or providing them with stable Wi-Fi, maximizing privacy using encrypted servers, and finding a suitable space for these meetings. Ensuring adequate engagement by keeping younger children interested through sending them hands-on material to engage with during web-based sessions seems desirable. In addition, medical providers require adequate training with sufficient space and knowledge of web-based learning tools. Finally, a couple of studies noted racial inequities in accessibility to mental health programs [48,51,52,74], highlighting that this needs to be addressed to ensure successful and equitable mental health program and service implementation.

## Discussion

### Principal Findings

The objective of this literature review was to broadly gain a better understanding of the types of programs that were implemented during the pandemic for child and youth mental health along with a better understanding of implementation challenges and knowledge dissemination strategies, including KT theoretical models for program implementation. This included clinical programs in health care settings and community-based programs in schools. It also included individual- and family-level preventive strategies for early identification and referral through knowledge dissemination in mental health training programs. The implications of the results of each aim will be discussed in this section.

First, we aimed to better understand the key barriers and challenges regarding implementing child and youth mental health programs during the pandemic. Certain factors need to be taken into account, including internet accessibility, to ensure that everyone has equitable access and that there are no technological barriers to timely mental health care for youth. Privacy was also raised as an important barrier in e–mental health among youth, highlighting that secure digital media need to be used. There were issues with Wi-Fi accessibility, emphasizing that a stable internet connection is vital for implementing these programs for youth. Policies could consider funding Wi-Fi for families who may struggle financially with respect to purchasing high-speed, stable internet plans. In addition, having a secure and private space is important for both the practitioners and the patients. However, it is challenging for patients to make room for meetings if they do not have adequate space at home. Nevertheless, from a program implementation stance, it appears that mental health services via telehealth are acceptable for young adults overall. However, it seems that younger teenagers and children may require additional strategies to avoid “Zoom fatigue” and keep them interested and engaged. For these reasons, the programs opted for offering hybrid care to youth or combined media such as apps along with family supportive services, whereas children received art kits or play kits to enhance participatory engagement.

Second, we aimed to better understand what types of mental health programs were implemented during the pandemic by key stakeholders and what KT theoretical models and strategies were adopted during the implementation of these programs. We identified a few initiatives, particularly in Canada and Australia, that worked toward providing mental health care to young adults while engaging with critical stakeholders. Important stakeholders included schools, medical doctors, psychologists, the criminal justice system, families, young adults with lived experience, social workers, charities, and politicians, among others. Some offered training to the stakeholders, and others provided direct mental health support to those with lived experience.

We identified several programs that were implemented for youth during the pandemic, with most being telehealth and digital based, followed by hybrid (mixed face-to-face with digital) and in-person face-to-face programs. However, overall, many programs recommended a hybrid method for delivering youth mental health services, including the use of digital media and face-to-face sessions to increase accessibility and meet the learning pretenses of stakeholders. The digital media included apps, the use of Zoom, and other web-based tools or websites.

Some the initiatives used clear KT theories to inform their programs, and 7 of them had used psychological theoretical models. There is a need for more implementation programs to use KT models when training practitioners to use evidence-based methods. Some programs also used theories of behavior change and psychological theories to inform their interventions, such as facilitating positive experiences through meaningful expression and engagement [37].

The use of diverse media for KT rather than one medium appears to be important. In their review of KT interventions for parental knowledge of all childhood health problems published until 2015, Albrecht et al [81] found that most studies used one medium. Thus, the use of digital media in an increasingly digitalized world appears to be an important move toward enhancing accessibility for youth mental health. The pandemic has especially highlighted the urgent need for the transition to web-based methods to enhance accessibility despite different levels of stakeholder acceptability. However, more research is needed to better understand whether the programs and hybrid models are actually effective in improving youth mental health as this was not evaluated in the implementation studies.

Another aim was to understand what mental health first aid training interventions were developed for increasing knowledge of mental health support and understanding any challenges regarding their implementation or adoption. We did not identify many such studies that were undertaken during this period. Initially, we identified 6 studies that were published during the pandemic period, with many finding increased perceived self-efficacy for providing mental health first aid, but not all led to changes in behavior, including the actual provision of mental health support to someone in distress [82-87]. However, after following up and closer examination, the studies were not actually undertaken during the pandemic period itself.

### Recommendations

On the basis of the knowledge synthesized from this literature review, several recommendations can be made for youth mental health promotion in the postpandemic era and for future pandemic preparedness. A future youth mental health KT tool may also be developed based on these recommendations.

1. Evaluate more hybrid models of mental health for KT among various stakeholders.
2. Reduce the barriers to implementing tele–mental health in youth by providing adequate technological access, Wi-Fi and stationary internet connectivity, and privacy protection.
3. Enhance staff training and preparedness for a future pandemic by having the equipment, knowledge, and skills in place.
4. Undertake more research on youth mental health for future pandemic preparedness and first aid training, including the barriers to and facilitators of effective KT and implementation.

**Figure 2:**
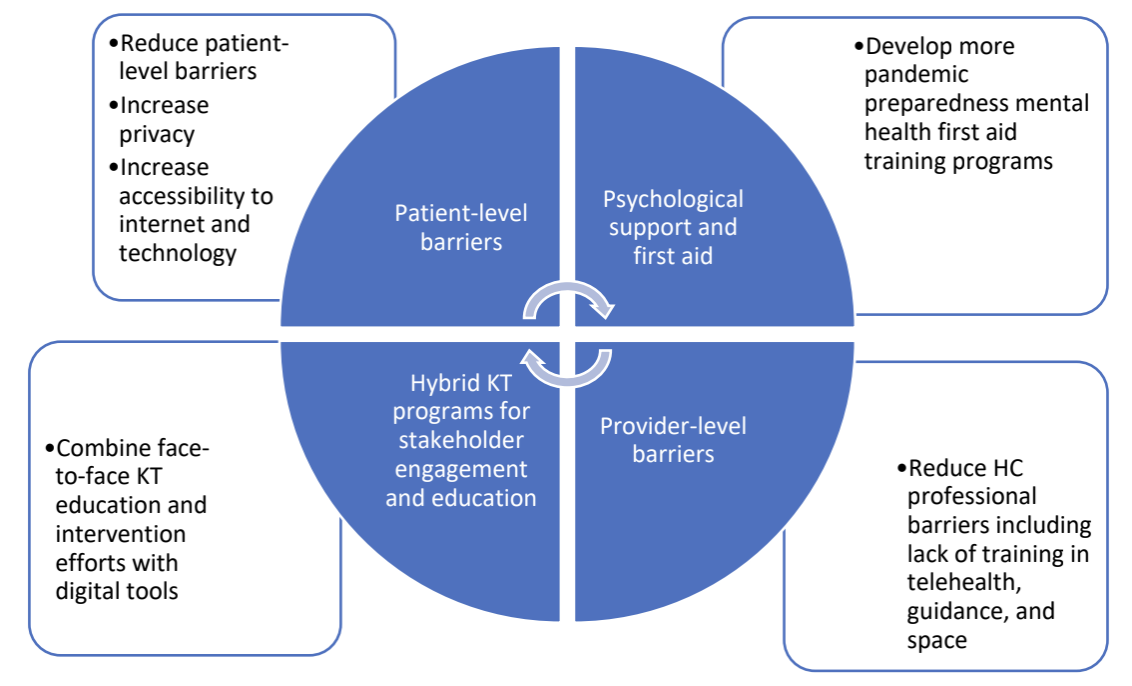
Future pandemic preparedness regarding pediatric and youth mental health. HC: health care; KT: knowledge translation.

Figure 2 illustrates 4 levels of steps that could be taken for future child and youth mental health and pandemic preparedness.

### Limitations

One limitation of this review is that we undertook more of a narrative literature review as, ideally, we would have required 2 screeners for a full scoping review, which was not possible due to resource limitations. We included publicly accessible free articles due to resource limitations. We also did not search gray literature, which could have provided more data on rapidly implemented studies that were not published. There is a possibility that there were more studies on this topic given the breadth of the COVID-19 literature.

However, our overarching aim was to gain a better understanding of the common implementation challenges and KT strategies that were developed in general during the pandemic, and we broadly covered the literature in several key areas spanning KT; implementation; school-based programs; and health care, including telehealth programs. A strength of this review is that we structured our analysis around KT and provided practical “hands on” recommendations for implementation and policy that may be applicable to many future studies, especially when planning for a future pandemic.

We note that there were wide variations in terms of countries and policies during the pandemic, but the overarching implementation issues were common across the studies despite this. In addition, although the studies are generalizable to pre- and postpandemic times, the focus was on the pandemic period to ensure that we understood what the challenges were during times of uncertainty and crisis, when swift decisions had to be made regarding new implementation issues.

### Conclusions

In summary, we aimed to better understand the implementation experiences, challenges, and facilitators of child and youth mental health program services during the pandemic. We found that, while many benefited from digital implementation strategies, hybrid in-person combined support was preferred. Provider-related challenges were also identified with transitioning to telehealth and learning how to use the technology. Barriers for patients were mainly privacy related and technological, including access to the internet and devices and the ability to communicate efficiently through a screen.

We also aimed to gain a better understanding of the KT intervention strategies, programs, and positive psychology interventions that were developed to promote youth mental health during the pandemic period. We identified KT programs that engaged with a wide range of stakeholders during the pandemic, and a few were KT theory informed. Future studies should focus on hybrid systems of KT and youth mental health program delivery and address technological and privacy barriers linked to the implementation stage of youth mental health e-services.

## Appendix

Multimedia Appendix 1 PRISMA-ScR (Preferred Reporting Items for Systematic Reviews and Meta-Analyses extension for Scoping Reviews) checklist.

## Abbreviations

KT: knowledge translation

## Data Availability

Data sharing is not applicable to this paper as no data sets were generated or analyzed during this study.
